# Situs Inversus, a Choledochal Cyst and a Horseshoe Kidney: A Strange Coincidence of Congenital Conditions

**DOI:** 10.7759/cureus.66757

**Published:** 2024-08-13

**Authors:** Nilanjan Sarkar, Rohit Chakravarty, Sandipan Mukhopadhyay

**Affiliations:** 1 Radiology, Tata Main Hospital, Jamshedpur, IND

**Keywords:** ct scan, ultrasonography, horseshoe kidney, choledochal cyst, situs inversus

## Abstract

Situs inversus is a condition in which abdominal and thoracic organs are laterally transposed. The organs which are supposed to be on the right side of the abdomen are on the left and vice versa. It is a rare congenital condition; however, the exact incidence is difficult to determine as most of the cases go unnoticed until they undergo an imaging study.

We report a case of a 30-year-old female presenting with situs inversus in association with a choledochal cyst and a horseshoe kidney. She underwent imaging evaluation for non-specific abdominal pain. Her routine clinical examination revealed a soft abdomen without any tenderness. Routine laboratory tests were within normal limits. Since there was abdominal pain, ultrasonography of the whole abdomen was advised. It revealed the presence of abdominal organs on the opposite side as normally seen. The common bile duct was dilated, and lower poles of the kidneys were fused. The cardiac apex was found to be on the right. It was followed up with a computed tomography scan which confirmed situs inversus. The common bile duct was dilated without any obstructive pathology in the pancreatic head or periampullary region. Lower poles of the kidneys were found to be fused together in front of the retroperitoneal vessels through an isthmus. Based on these findings, a diagnosis of situs inversus in a case of a choledochal cyst and a horseshoe kidney was made.

Situs inversus is a rare entity. Its association with choledochal cysts and horseshoe kidneys has never been reported in the literature to the best of our knowledge.

## Introduction

Situs inversus, choledochal cysts, and horseshoe kidneys are all congenital conditions and are rarely seen during clinical practice. The presence of all three conditions in a single individual is so rare that it has never been reported in the literature.

Situs inversus is a congenital condition and usually diagnosed incidentally during imaging studies which reveal thoracic and abdominal organs located in reverse order across the midline. Although it is asymptomatic by itself, detailed anatomical information may be required prior to any surgery. Imaging evaluation is required prior to any planned surgery [1].

Choledochal cysts are also congenital malformations in which the biliary system is dilated without any obvious obstructive pathology. There may be isolated or a combination of intra and extra-hepatic biliary dilation. In contrast to situs inversus, choledochal cysts may be symptomatic. The classical triad of presentation is abdominal pain, obstructive jaundice, and right upper quadrant abdominal mass but it is rarely found. Abdominal pain is most frequently observed in adults, whereas jaundice is the most common presenting symptom in children [2].

The horseshoe kidney is a renal fusion anomaly. Normally the kidneys are located wide apart across the midline in the retroperitoneum in the lumbar regions. However, there may be a congenital fusion of its lower poles through an isthmus. Their normal axis is altered and fused kidneys look like a horseshoe [3].

We present a case of a choledochal cyst and a horseshoe kidney in a 30-year-old female with situs inversus. It was diagnosed in an abdominal ultrasound scan followed by a CT scan of the abdomen.

## Case presentation

**Figure 1:**
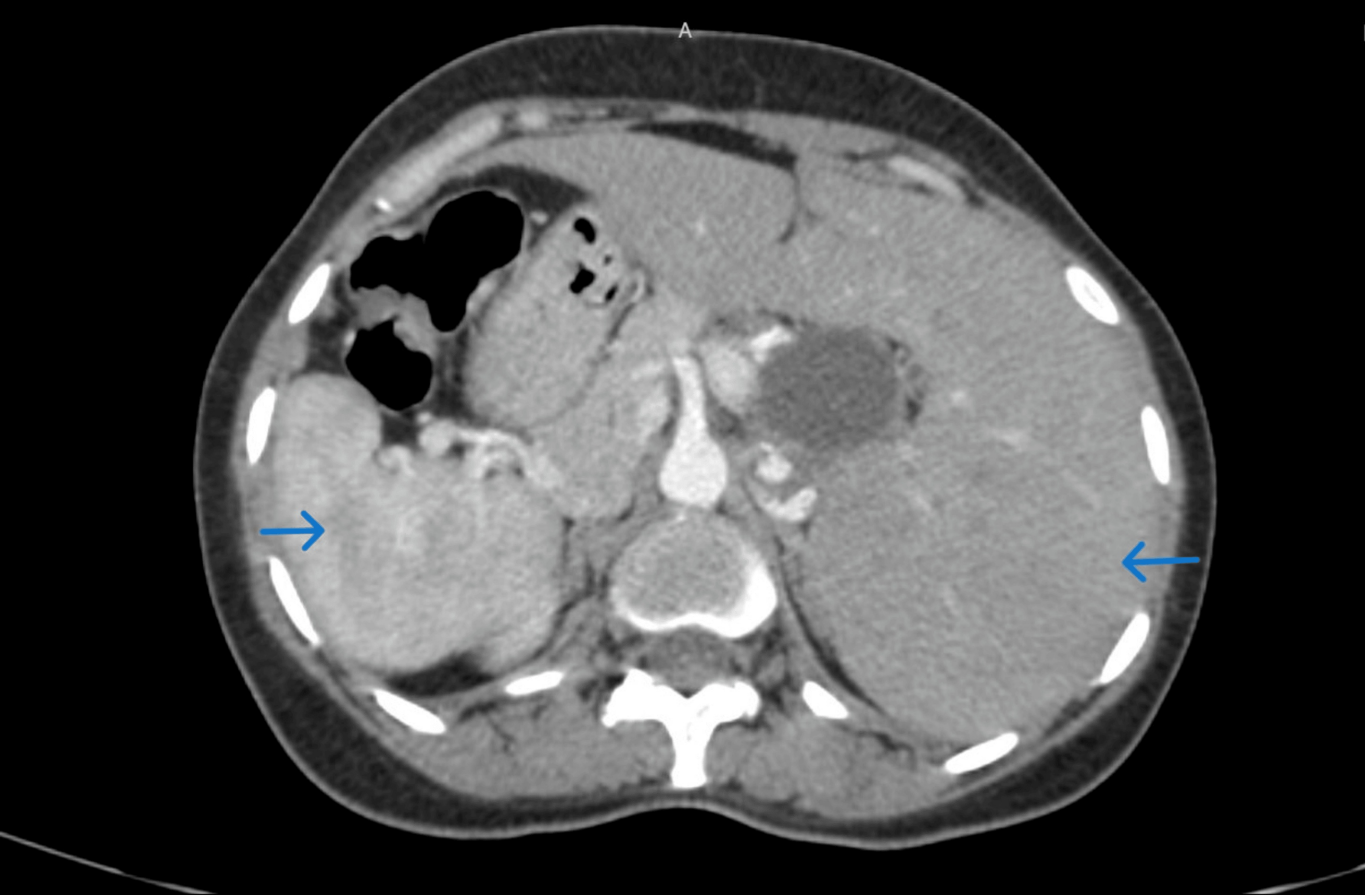
Axial late arterial phase contrast CT scan image of the abdomen shows the spleen and stomach on the right and liver located on the left side of the abdomen. The choledochal cyst was also visualized. CT: computed tomography

A 30-year-old female presented with intermittent pain abdomen and defecation difficulty for a few months. The pain was non-specific and localized mainly in the region of the epigastrium and right hypochondrium. On examination, she was afebrile. Her build was average. There was no pallor. No clubbing or edema was also noted. Pulse was regular and blood pressure was well within normal range. The abdomen was soft without focal tenderness. There was no weakness in extremities or signs of focal neuro-deficit. Routine blood parameters showed normal liver function tests. Hematological and renal parameters were normal. Considering the presence of pain, she was advised ultrasonography of the whole abdomen. It revealed the presence of abdominal organs in positions opposite to normal locations in a mirror-image fashion. The subxiphoid view revealed the presence of a cardiac apex on the right side. The liver was enlarged with normal echotexture. The common bile duct was dilated along the entire length and there was no calculus or sludge in the visualized part of the lumen. Lower poles of the kidneys were fused. A contrast CT scan of the whole abdomen was advised especially to see the periampullary region for which ultrasonography is of limited value. It revealed juxtaposition of abdominal organs (Figure 1).

**Figure 2:**
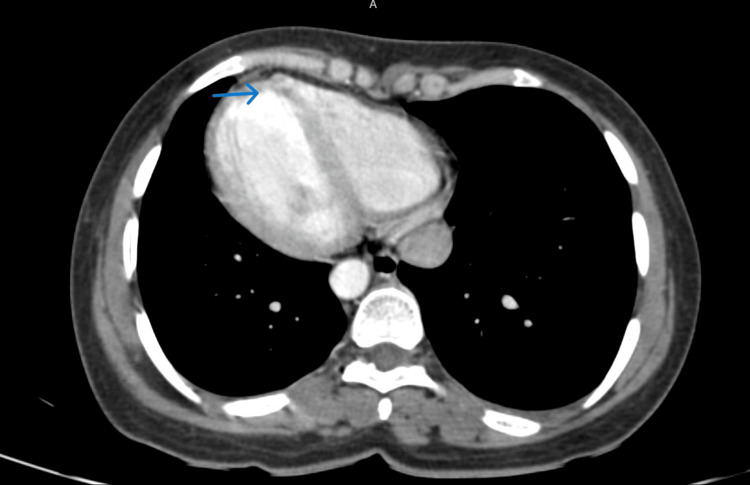
Axial late arterial phase contrast CT scan of the abdomen in lower thoracic sections shows the cardiac apex on the right side CT: computed tomography

In the lower thoracic images, the cardiac apex was seen on the right side (Figure 2).

**Figure 3:**
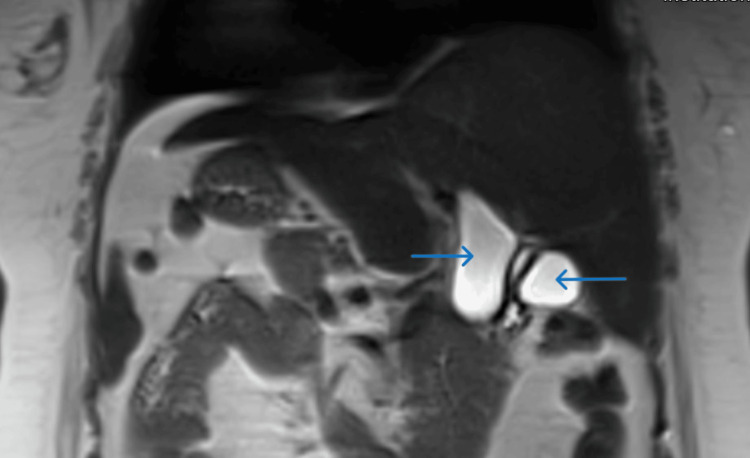
MRCP shows the choledochal cyst and gallbladder MRCP: magnetic resonance cholangiopancreatography

There was dilation of hepatic ducts and common bile duct till the terminal end. The diameter of the common bile duct at porta was 37mm. There was no dilation of intrahepatic biliary radicles as well as the main pancreatic duct. No mass lesion was detected in the head of the pancreas or periampullary region. Subsequent magnetic resonance cholangiopancreatography (MRCP) confirmed these findings (Figure 3).

A diagnosis of type Ia choledochal cyst was made as per the Todani classification [4]. There was no feature of cholangitis.

**Figure 4:**
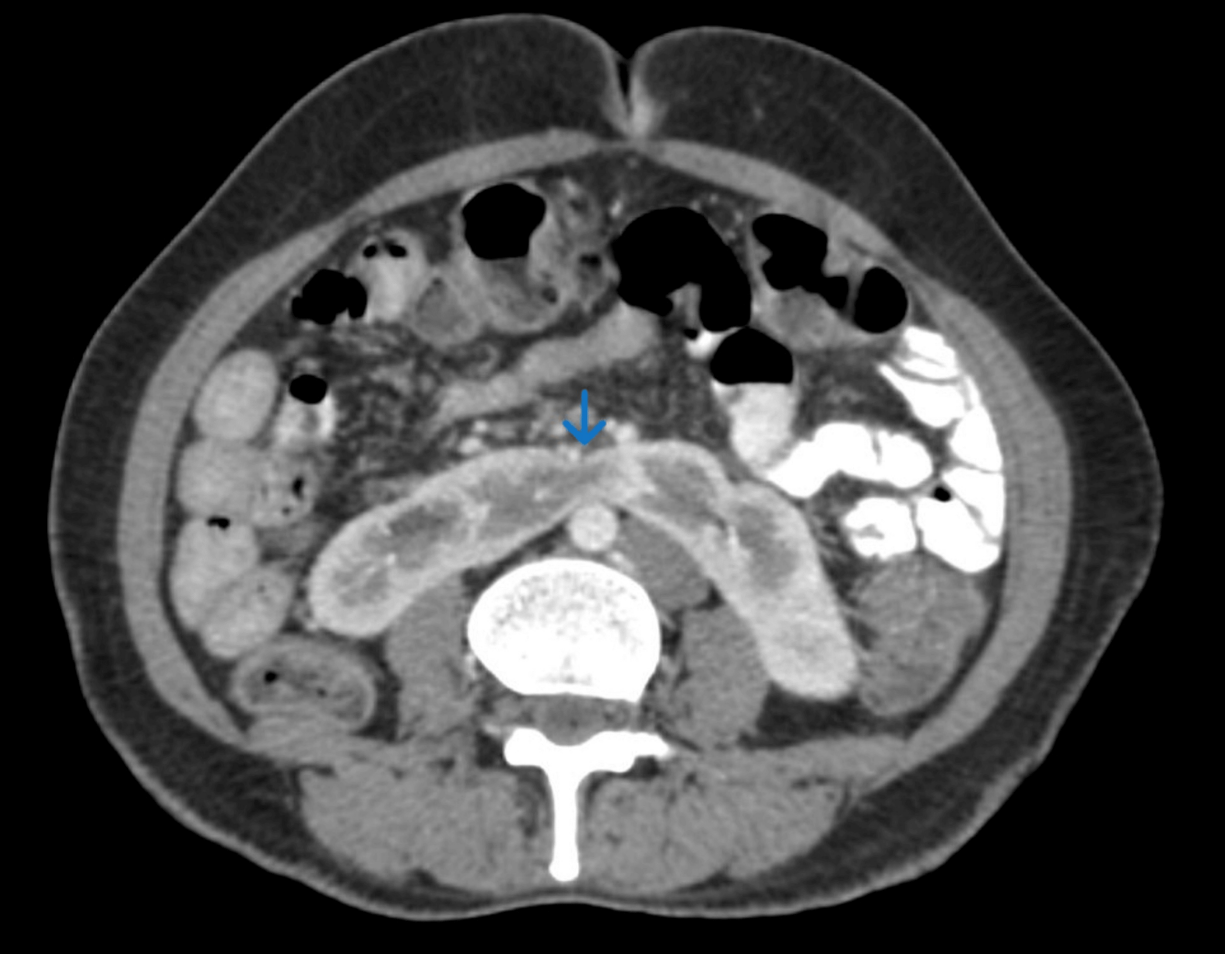
Axial late arterial phase contrast CT scan of the abdomen shows the horseshoe kidney CT: computed tomography

The presence of horseshoe kidneys was confirmed with isthmus across the midline in front of the retroperitoneal vessels (Figure 4). Kidneys were located below their normal anatomical position. The pelvis of the kidneys was located anteriorly with mild fullness.

Based on these findings, it was diagnosed as a case of situs inversus totalis with the choledochal cyst and horseshoe kidneys.

Surgery was advised for the presence of the choledochal cyst which she refused. At one-year follow-up, she was asymptomatic. 

## Discussion

The normal position of thoraco-abdominal organs which is present in the majority is called situs solitus. Situs inversus is a condition in which thoraco-abdominal organs are in a position just opposite to the normal anatomical location in a mirror-image fashion. The term situs inversus totalis is used when major abdominal and thoracic organs are completely reversed in position. Some studies suggest its prevalence to be 0.01% of the whole population [5]. It is usually asymptomatic per se as there is no physiological variation in the concerned organs. However, surgical and interventional procedures become difficult considering that most of the surgeons and radiologists are right-handed [6]. The operator will have to stand opposite to the traditional side.

Determination of side is dependent on the expression of certain genes like Nodal and PITX2 and it happens early in the development typically during gastrulation [7]. Situs inversus is mostly found in isolation; however, it may be part of syndrome, most commonly “primary ciliary dyskinesia” [8]. It is known as Kartagener syndrome consisting of situs inversus with bronchiectasis and sinusitis.

Choledochal cysts are an uncommon congenital condition where there is dilation of extra or intrahepatic bile ducts or a combination of both. They usually present in childhood with features of abdominal pain, abdominal mass, and jaundice. The incidence of diagnosis of choledochal cysts is much higher in the Asian population with an incidence of 1 in 1000 live births and more frequently seen in women. In the Western population, it is relatively rare, seen in 1 in 100000 individuals [9]. Radiological imaging reveals intrahepatic and/or extrahepatic biliary dilation without any obvious cause like calculus or mass lesion in the biliary tree, pancreatic head, or periampullary region. The primary investigation is ultrasound which confirms biliary dilation. A CT scan or MRCP is advised to confirm these findings as well as to rule out any neoplastic pathology in the periampullary region or pancreatic head. Although having high sensitivity and specificity ERCP is of limited use due to its invasive nature and risk of ionizing radiation. Surgery is advised considering risks of malignancy, cholelithiasis, cholangitis, and pancreatitis [10].

Horseshoe kidneys are congenital fusion anomalies with an approximate incidence of 1 in 500 individuals [11]. In its most common variant, the kidneys are located on each side of the midline with their lower poles fused through an isthmus. The isthmus consists of renal parenchyma and it has been proposed that abnormal migration of nephrogenic cells is responsible for this ectopic mesenchymal tissue. Relation with some genetic and autosomal disorders has also been noted [12]. Usually, horseshoe kidneys are asymptomatic conditions but they may present with hydronephrosis, renal calculus, infection, and neoplasia. They are also more prone to traumatic injury. Pelvi-ureteric junction obstruction is very commonly associated with horseshoe kidneys [13].

To the best of our knowledge, this is the first reported case where there is situs inversus, a choledochal cyst, and a horseshoe kidney in a single patient and needs further research to find out any related genetic condition.

## Conclusions

Situs inversus, choledochal cysts, and horseshoe kidneys are congenital conditions. Except for choledochal cysts, the rest are usually asymptomatic and diagnosed incidentally during imaging studies. Choledochal cysts are a potential risk factor for malignancy and surgery is usually recommended. Any surgical or intervention radiology procedure is challenging in patients with situs inversus due to altered anatomical orientation with which operating doctors are not habituated. These conditions are rare and their presence together in a single individual has never been reported in the literature. To date, no genetic or embryological reason has been found for them to present together. Radiological investigations play a pivotal role in their diagnosis. Ultrasonography can provide baseline information; however, cross-sectional imaging studies like CT scans can provide detailed anatomical information regarding the malformations as well as rule out any associated pathology.
